# EEMIP: Energy-Efficient Communication Using Timing Channels and Prioritization in ZigBee

**DOI:** 10.3390/s19102246

**Published:** 2019-05-15

**Authors:** Pavol Gočal, Dominik Macko

**Affiliations:** Faculty of Informatics and Information Technologies, Slovak University of Technology in Bratislava, Ilkovičova 2, 842 16 Bratislava, Slovakia; gocal.pavol@gmail.com

**Keywords:** energy-constrained device, energy efficiency, Internet-of-Things, low-power communication, wireless sensor network, ZigBee

## Abstract

With the expansion of the Internet-of-Things, energy-efficient communication is becoming vital. The communication among energy-limited devices (e.g., powered by batteries or harvesting the energy from their environment) must be energy-efficient, prolonging their lifetime or increasing data throughput. This article aims at proposing energy-efficient periodic communication for devices over the ZigBee protocol and powered by a battery. We propose using timing channels for different data priorities, thus, more important data are sent more frequently. The priority is also considered in case of congested traffic, where a central device (coordinator) prioritizes more important communication. We have implemented a simulator, which serves for verification of the proposed solution, and conducted experiments comparing the proposed EEMIP method with the standard nonbeacon ZigBee communication. The experimental results show that the proposed method is more energy efficient.

## 1. Introduction

Requirements for reducing energy consumption in devices are still increasing due to population growth and industrial development. The problem of the energy consumption is one of the key aspects of Internet applications [1]. Devices with limited power supplies that are part of the Internet-of-Things (IoT) should have optimized interfaces, and optimized communication protocols are expected to reduce energy consumption. This means that when two or more interconnected devices communicate with each other, you will not need to change batteries or charge devices after a short while. Based on several factors (e.g., data characteristics, data transfer mode, network size), there are still lots of possibilities to optimize protocols based on the network needs.

Energy efficiency is especially important for end sensory devices with limited energy sources, which are connected by means of so-called wireless sensor networks (WSN). The WSNs, thus, create a periphery of an IoT domain, which interacts with the environment. In WSNs, there is a number of cases in which the device topology contains a central node that collects and evaluates acquired data from sensory devices, such as parking cameras [2] or hospital equipment [3]. There may be a case in which the network traffic and communication are too high and the central node does not manage to process all the data. The reason may be the production of unnecessary communication or periodic transmission of data from sensory IoT devices at very short intervals. As a result, some data may be lost.

There are several studies that deal with the reduction (or efficient use) of energy for devices in the WSN or IoT areas. For example, a decent survey on energy efficient techniques in WSNs is provided in [4]. It identifies five main classes of these techniques: data reduction, protocol overhead reduction, energy efficient routing, duty cycling, and topology control.

The data reduction usually deals with compression or aggregation of data to minimize the amount of transmitted and processed data. However, there are also approaches to reduce the amount of data produced by sensors by using various sampling techniques [5,6], such as adaptive, hierarchical, event-triggered, or model-based sampling. The ADAPT (ADaptive Access Parameters Tuning) framework [7] offers optimized data collection in WSNs based on IEEE 802.15.4/ZigBee standards. It enables to adapt media access layer configuration according to the application’s reliability requirements and the traffic conditions in order to minimize power consumption. Authors of [8] proposed an event-triggered IoT communication. For saving power, IoT devices often support switching between some active and passive (i.e., sleep) operation modes. However, in many cases, periodical switching between these modes leads to unnecessary activation of the device, which decreases its energy efficiency. IoT communication based on events solves such an issue, since the device is activated only after some event occurs and it, thus, generates less data for transmission. Such an approach is very effective especially for monitoring and controlling purposes. A similar approach was proposed in [9], which utilizes cellular networks for sending a wake-up signal to an IoT device to switch it to an active mode. Event-triggered communication was also used in [10], which proposed two innovative medium access protocols for decentralized control systems.

In [6], there are also various media access protocols discussed, such as B-MAC, STEM-T, WiseMAC, S-MAC, or UBMAC. These protocols already target ultra low power operation. In [11], the authors have reduced control overhead of S-MAC protocol and proposed an optimized LO-MAC protocol. Various energy efficient routing techniques and strategies are surveyed in [12]. The authors compared multiple energy-efficient WSN routing protocols, such as LEACH, HEED, DECA, SPIN, and PEGASIS, and identified as their main weakness an assumption that the nodes are static and stationary.

In [13], the authors focused on the ODMAC protocol, which serves to reduce the energy consumption of devices at the data link layer. The reduction of energy was achieved by so-called duty cycles, which are specific for each device. The ODMAC was designed on the basis of three key objectives: sustainability, efficiency and application performance. Devices using ODMAC adapt their duty cycles based on ENO (Energy Neutral Operation); the node is sustainable, if for a certain period of time, the energy consumed is less than or equal to the energy harvested. All nodes in the network dynamically set the beacon frame and modify duty cycles to obtain maximum performance status. This means that when the energy used is higher than the energy obtained, the duty cycles are reduced to reduce energy consumption. The authors of [14] proposed dividing BLE (Bluetooth Low Energy) communication into time channels. Information is divided into time intervals. Improving energy efficiency is achieved by adding additional information at the time of inactivity between sending BLE packet advertisements. Added bits are encoded without increasing work cycles, thus reducing BLE transmitter power consumption. It has been proven that usage of time channels, in which information is sent at intervals, will reduce energy consumption and increase energy efficiency by more than 10%. There is a number of other approaches developed to utilize duty cycles in the communication to minimize energy consumption [15], which use various ways to synchronize devices and to enable them to be put into a power-saving state for some time period.

The last class of energy efficient techniques is based on a topology control. The key idea is to create and maintain a reduced topology while preserving the original connectivity and coverage. In this class, there are also techniques that use a distributed environment of WSNs to reduce overall energy. An approach, proposed by [16], utilizes proactive planning in sharing Internet connection among multiple IoT devices. IoT already enables to discover devices and create a communication connection among them autonomously. It is beneficial not only for sharing information, but also for sharing Internet access, which can save energy. This way, the proposed framework proactively plans Internet services in a cooperating IoT environment. In [17], the authors focused on an energy-saving solution using ZigBee devices in IoT. The idea was based on the fact that the IoT domain has more and more devices that are capable of computational operations. Devices could use the processing capabilities and calculations of other devices to achieve the desired goal. One of the main aspects was to mediate network collaboration. ZigBee devices usually communicate on a local network and are not connected to the Internet. As a solution of this problem, the Internet-of-Things Group Distributed Computing Platform (IGDCP) was proposed. IGDCP includes IoT devices and devices connected to the local network.

In general, IoT connects heterogeneous devices, communication technologies, sensors and transferred data. To increase efficiency, shared gateways provide Internet connections for such heterogeneous technologies and data. However, some data are critical and others are not; therefore, a system should provide some mechanism to minimize latency or increase reliability of delivery of critical data. It means that there is an increasing importance to incorporate so-called quality of service (QoS) and implied prioritization into IoT sphere [18,19,20]. Another way of dealing with heterogeneity in IoT is to use some 5G network technologies [21,22], such as NOMA (NonOrthogonal Multiple Access) [23]. NOMA increases spectral efficiency by enabling simultaneous communication of nodes and using power differences and successive interference cancellation to demultiplex the signals. It enables us to use different bandwidth/power for different communication nodes. It is more efficient for networks with a large number of devices with a high variety of communication requirements. However, there are applications for which the TDMA (Time Division Multiple Access) based on duty cycles is more efficient [24].

We have analyzed the advantages of the existing works and have been inspired by them in our proposal of a modification of the basic ZigBee protocol. ZigBee was selected because of its small control overhead compared to other similar technologies, such as BLE. We have combined the duty-cycle-based approach with prioritization at the central node of ZigBee star-topology communication. The goal was to increase energy efficiency of periodic sending of end-node sensor data to the central node by reduction of simultaneous access to the medium and resulting retransmissions. This way, more important data are preferred over less important, and they are not lost in case of a traffic congestion. The solution is designed for the star WSN network topology with one central device (an Internet gateway, also called an IoT gateway) and several end sensory devices, which can use various data priorities (e.g., multiple sensors in a single end device with different data). The proposed method is called EEMIP (Energy-Efficient Method using Intervals and Prioritization). We intend to use the method for health-monitoring applications, but the method can be used for other applications as well.

The article is structured as follows. In this section, we have summarized motivation and similar studies that deal with the reduction of energy in communication. In Section 2, a background regarding ZigBee technology is given. Section 3 contains proposal of a new method for energy-efficient communication targeting ZigBee protocol. In Section 4, the experimental results are described and discussed. The last section concludes the article.

## 2. Background

ZigBee [25] is a wireless personal area network (WPAN) technology targeting low data rate applications requiring low power operation. It supports multiple network topologies, specifically star, mesh, and cluster tree. It is especially useful for low-power IoT applications, since it requires relatively small amount of energy for data transmission, which enables the connected devices to run several year on batteries (for comparison of energy efficiency of several technologies, see [26]). There are three types of logical devices in ZigBee [27]: coordinator, router, and end device. A coordinator creates and manages the network, it must be aware of all the connected devices. A router is an intermediary device, which forwards traffic between the coordinator and other devices that are not directly connected. An end device is a reduced function device (usually battery operated), which interacts with the environment using sensors/actuators, it cannot forward traffic between other devices. In the star topology, the coordinator is usually a central node, which must be a full function device.

ZigBee supports three data-transfer types [28]: from a device to the coordinator, from the coordinator to a device, and between two devices. The exchange of messages depends on whether a beacon frame is used for synchronization or not (there are beacon and nonbeacon modes available). A beacon mode is usually considered more power-efficient [26], since it allows the end devices to switch to a sleep mode for some period between individual beacon frames. However, it also requires the devices to wait and listen for such a beacon frame and afterwards wait for their time slot to communicate (uses the slotted CSMA/CA for a channel access), and thus waste power. In other words, a device must be awake (i.e., not in a power-saving state) for a time *T*, which is given by the following simplified equation:

(1) T=Process+Wait_beacon+Wait_slot+Transmit+Wait_ack,

where *Process* represents processing time (generating data to be sent), *Wait_beacon* is a variable time period, for which the device must wait for the beacon frame after the data are prepared, *Wait_slot* represents the period the device is waiting for its time slot (which might be guaranteed or the device must compete for nonguaranteed time slot that might include back-off time in case of multiple devices trying to communicate simultaneously), *Transmit* is time required to send the data, and *Wait_ack* is time required to wait for the Acknowledge message in case of a reliable delivery. It must be noted that the retransmission can be required if the Acknowledgement message is not received, which prolongs the device’s active time. If there are multiple kinds of devices with various sensors in the network, some of which require sending data more frequently than the others, the beacon mode makes them to unnecessarily (i.e., no data to send) process the beacon frame, or even wake up to receive it, which wastes more power.

Nevertheless, the duty-cycle approach itself has undeniable potential in energy savings. There is, however, need to use different duty cycles for different devices in the same ZigBee network, and thus, minimize their waiting period, during which the devices must be awake. A synchronization method that is different from the beacon mode must be used to exchange data between the coordinator and multiple end nodes. Ideally, the device would be active only for processing time, transmission time, and waiting for acknowledgement that is always received (no retransmission required). The optimal active time To would then be expressed by the following equation:

(2) To=Process+Transmit+Wait_ack.

This is achievable if the end device periodically generates the same amount of data (this is the case of most sensory devices), since the processing and transmitting time is predictable. The device can then wake-up just before its time slot; enough to prepare data for transmission.

## 3. The Proposed EEMIP Method

**Figure 1:**
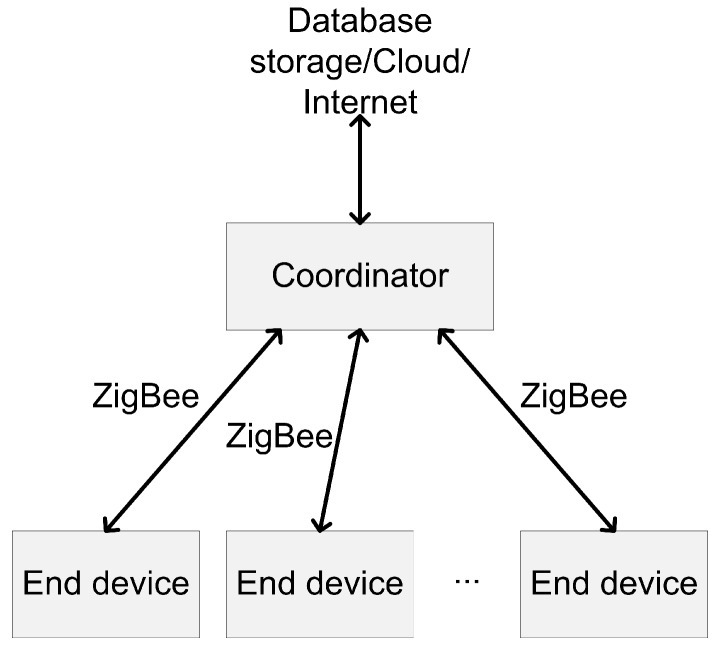
Overview of the considered architecture.

Based on the state-of-the-art, we propose a solution (i.e., the EEMIP method) to increase energy-efficiency of periodic ZigBee communication with a reliable delivery. In the proposed solution, we use two main nodes in the network topology; one coordinator, which controls the communication flow, and multiple end devices with sensors. Since we target the star topology, there is no need for router-type devices. The two types of nodes communicate with each other in order to exchange data. An architecture overview is provided in Figure 1.

The key idea is to use a nonbeacon ZigBee mode, but each end device is assigned with a unique time slot for communication. Based on the different priorities, the end devices use different time periods, which enable some devices to communicate more frequently than the others. It is also useful in a case of traffic congestion, where the coordinator ensures unaffected delivery of high-priority data (e.g., hearth failure, fall) in contrast to low priority data (e.g., temperature, pulse), which may be delayed. The method is described in the following subsections in more detail.

### 3.1. Design Requirements

We have stated several requirements, which the proposed solution should meet:

- *Using end devices with sensors*—the end devices can have different sensors, producing different amount of data. A single end device can have multiple sensors.
- *Using central device for data collection*—the central device (coordinator) collects data from end devices and can send them for further analysis or usage to an external device (e.g., via Internet connection).
- *Using energy-efficient communication*—an energy-efficient communication is required for devices with a limited power supply. Therefore, the network nodes use the proposed method for communication.
- *Using priority for data*—more important data are sent more frequently than less important data and are preferred in a case of congestion.
- *Using time intervals for data*—based on data priority, different time intervals are assigned to devices and each device communicate in a dedicated time slot.

### 3.2. Messages Types

The nodes in the ZigBee network use three new messages for communication:

- *Control message-Offer*—the message includes a number of available priorities. The higher the number, the lower the priority—0 is not an option, 1 is the highest priority, 7 is the lowest priority.
- *Control message-Selection*—the message includes selected priority for data.
- *Data transmission message*—the message contains priority, sensor type, and data.
- *Acknowledgement*—the message in the original ZigBee protocol, sent to inform the end device that data have been received.

**Figure 2:**
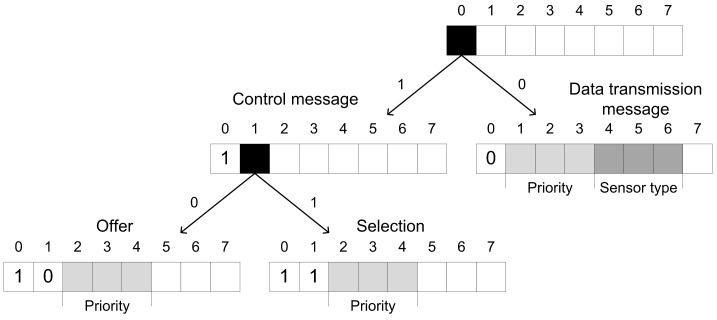
Bitwise distribution of message types.

Bitwise distribution of message types is illustrated in Figure 2. Based on the first received bit it is decided whether it is a Control message or Data transmission message. In case of the Control message, the second received bit differentiates Offer and Selection messages.

### 3.3. Control Messages Exchange

**Figure 3:**
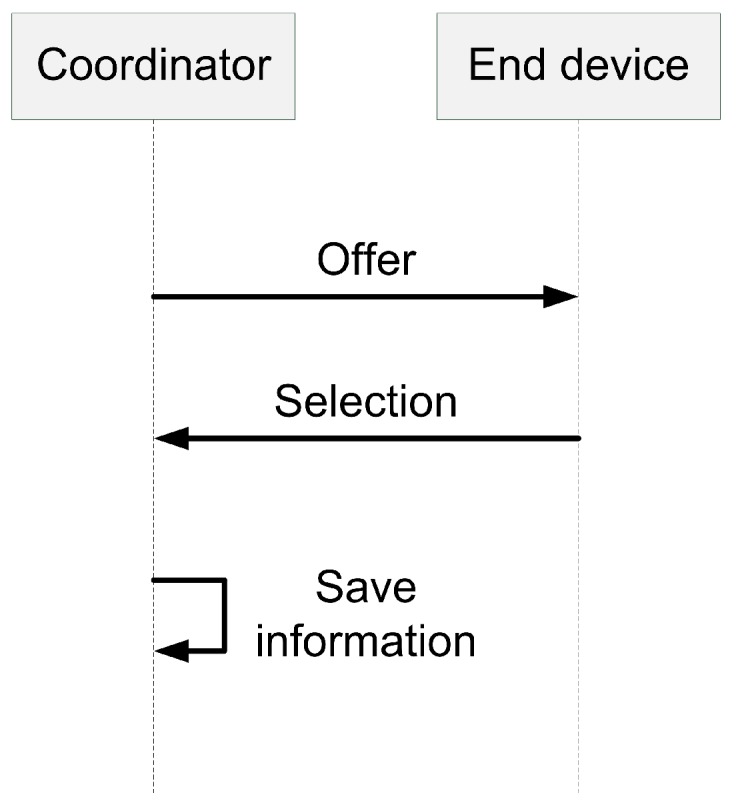
Exchange of control messages.

The coordinator and an end device use a simple model for exchanging messages. For offering available priorities in a network, the coordinator sends the Offer message to the end device, which contains priorities it can use. The end device receives the Offer message and replies using the Selection message with the selected priority for its data. The coordinator receives the reply and stores the information. This process is illustrated in Figure 3.

### 3.4. Priority and Time Interval Selection

**Figure 4:**
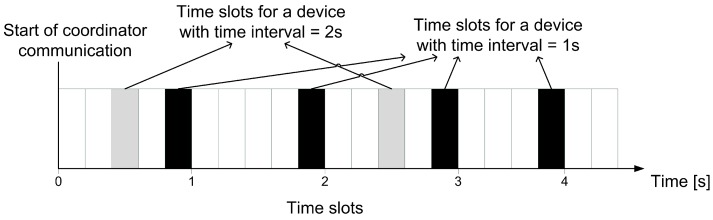
Time slots and time intervals example.

Before the data transmission, the end device needs to decide which priority is used for specific data. This process is explained using an example of the prototype. The prototype of the proposed method supports two priorities, low and high. An end device can assign more important data with the high priority. Based on the selected priority, the time interval is set for the data flow. The time interval is a period in which the device starts the communication (e.g., a time interval of 5 s means that each five seconds the device sends its data). The coordinator can communicate with end devices only during free time slots and cannot communicate with multiple devices at the same time (i.e., each device has a dedicated time slot). Based on this fact, the end device uses time slots to compute its time interval for transmission. Time intervals are computed based on a timestamp from communication with the coordinator. To clarify the difference between a time interval and a time slot, we provide an illustration of time slots in Figure 4.

The time interval selection algorithm of the end device is provided in Algorithm 1. The algorithm for a time interval assignment by the coordinator to an end device is provided in Algorithm 2.

**Algorithm 1:** Time interval selection and operation algorithm for an end device. **Data**: ZigBee communication-initiation phase is over. Time intervals *TI* for priorities are configured. **Input**: Received Offer message at the time *T*. **Output**: Selected time interval *TI(P)*.1 application selects a suitable priority *P* from the Offer message2 **if** Selection not sent **then**3  send Selection with priority *P*4 *Sleep time* = (*TI(P)* − time required for data processing)5 sleep **while** time < (*T* + *Sleep time*)6 **repeat**7  process data8  send data9  wait for Acknowledgement10   sleep for (*Sleep time* − time actually waited for Acknowledgement)11 **until** another Offer message received

**Algorithm 2:** Time interval assignment and operation algorithm for the coordinator. **Data**: Time intervals *TI* for priorities are configured. **Input**: Received message from the device *D*. **Output**: Assigned time interval *TI(P)* for the device *D*.1 ZigBee communication initiation of device *D*2 **if**
*D* not assigned with time interval **then**3  send Offer message with available priorities to *D* at the time *T*4  wait for Selection message from *D* with priority *P*5  assign *D* with time interval *TI(P)* based on the time *T*6 **repeat**7  wait for data from *D* in time interval *TI(P)*8  send Acknowledgement to *D*9 **until**
*D* communicates out of time interval *TI(P)*

The example of time interval selection is as follows.

*Premises*:

- Two types of priorities—high and low
- The high priority time interval is 1 s
- The low priority time interval is 5 s
- Each end device has one sensor with one data type

*Steps included in time-interval selection*:

1. The coordinator sends the Offer message to an end device.
2. The end device receives the Offer message at the system time of 08:05:13. It recognizes that it is a free time slot; thus, it can start sending data based on this timestamp.
3. The end device selects high priority (1) for its data, replies with the Selection message, and starts transmission of its data in the time interval of 1s starting in 08:05:14 (+1 s because of delay).
4. Meanwhile, the coordinator receives the Selection message containing the selected priority from the end device. The coordinator stores the information, so it is aware of the new device.
5. The coordinator receives data from the end device and sends the Acknowledgement message back to the end device.

Since the coordinator is coordinted with all the end devices in the network, it can recompute and identify free time slots when a new device arrives. The end devices synchronise when they can communicate, so there is no wait period for any synchronization (beacon) frame, nor for time slot (in a sense of standard beacon mode ZigBee communication). Therefore, the end devices can spend most of their lifetime in a sleep mode and wake-up only for their dedicated time slots (without wasting power for waiting). If the coordinator identifies that some end device is getting desynchronized, it can re-initiate its time-interval selection procedure, which resynchronizes it again, by resending the Offer message. Because of centralized management by the coordinator, the communication collisions are not really an issue (they occur only for a short period of time when a new device is not yet assigned with a dedicated time interval and not synchronized with others). However, the coordinator, as a single processing node, could get congested by the traffic from too many end devices. In such a case, the priority-based selection of time intervals has other uses, specifically, to ensure that high-priority data are not lost. Some of the low-priority data are sacrificed in order to deal with the congestion. As a result, less retransmissions occurs, since high-priority data are sent more frequently. This increases energy efficiency even more.

## 4. Results and Discussion

To verify the energy efficiency of the proposed method for ZigBee communication, we have implemented a network simulator. The simulator has two operating modes which it can simulate:

1. Nonbeacon ZigBee communication,
2. Communication using the proposed energy-efficient method (EEMIP).

**Figure 5:**
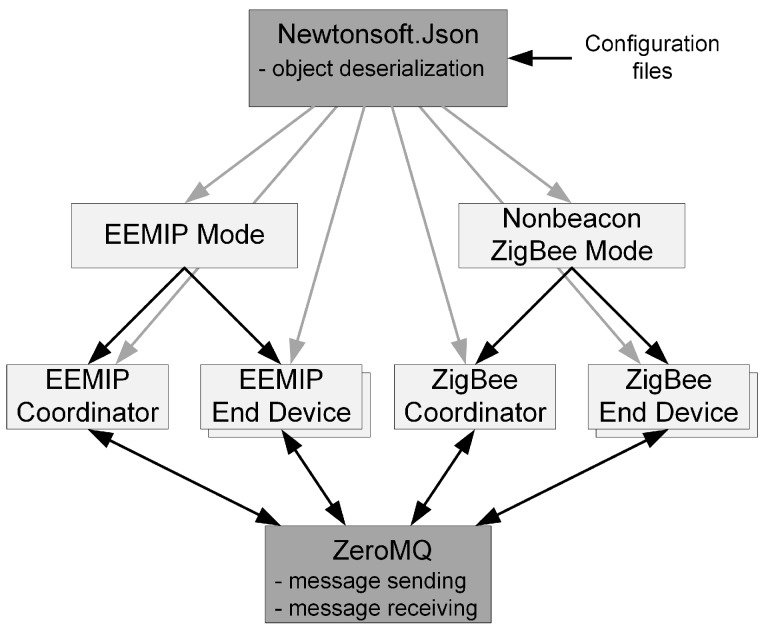
Simulator architecture overview.

The nonbeacon ZigBee mode has been selected because it is more efficient (regarding data transmission itself) than the beacon mode (the end devices have to process beacon frames, which consumes power). The simulator was implemented as a console application using. Net framework and Windows operating system. The architecture of the implemented simulator is illustrated using a block diagram in Figure 5. Dark grey color represents the used external libraries. The Newtonsoft.Json library [29] has been used for loading the configuration files. For communication between the coordinator and end devices, sockets operations using the ZeroMQ library [30] have been utilized.

As part of the simulation, we assume that the network initialization has already taken place, i.e., the ZigBee network is created by the coordinator and all end devices are connected to the network and ready to send data. We are not simulating creation of a connection or exchanging initialization messages, since this is not important from our point of view. Our method aims at energy-efficient communication in the data transfer stage.

Simulation runs in both available modes. For each mode, we send the same data and the simulation runs for the same time period (e.g., 1 h). The data to transfer for each end device can be specified in the configuration file. The separated configuration file is available for all end devices and the network coordinator in JSON format. The configuration file for the coordinator includes a number of nodes in the network, a number of different priorities, and time intervals for individual priorities. There is also a single global configuration file, which contains these configurable parameters:

- *Test mode*—determines whether the simulation is run until stopped or just the predefined time period (e.g., 1 h).
- *Simulation mode*—determines whether standard nonbeacon ZigBee mode is used or EEMIP.
- *Congestion simulation*—determines whether a congestion is simulated.
- *Data autogeneration*—determines whether the data for transmission will be pseudorandomly generated or data defined in the configuration file are used.
- *Congestion packet ordinal number*—determines which packet will be lost in case of congestion simulation (e.g., each 5th packet).
- *Low priority time interval*—defines a time interval value in milliseconds for low-priority data.
- *High priority time interval*—defines a time interval value in milliseconds for high-priority data.

Using various parameter values in the global configuration file, we can run simulations in various modes with various functions.

The EEMIP mode retransmits lost messages (i.e., Acknowledgment was not received) differently than the nonbeacon ZigBee mode. The nonbeacon ZigBee mode resends the message three times at maximum (according to Zigbee specification [25]). An end device waits for the Acknowledgment message from the coordinator for a time period *T_ACK_*, given by the equation:

(3) $$T_{ACK}=0.05*\left(2*nwkcMaxDepth\right)+\left(securityencrypt/decryptdelay\right),$$

where (*securityencrypt*/*decryptdelay*) = 0.1, and *nwkcMaxDepth* = 15 (according to [31]). The result after using the equation is that the end device waits for the Acknowledgement message for 1.6 s. The EEMIP mode does not use separate retransmission messages, but concatenates the unacknowledged data to the next data in the next time slot dedicated to the end device (this is also done maximally three times). Thus, the number of messages is not increased and the overhead is reduced.

Energy efficiency is evaluated based on a number of transferred bytes in both modes. This information is written into the statistical files for each end device. Afterwards, we compare the obtained values and evaluate the results. We have tested three scenarios; Scenario A for nonproblematic conditions (i.e., without retransmissions), Scenario B for congestion simulation, and Scenario C for realistic conditions (i.e., 20 end devices, data autogeneration, occasional retransmissions). For the first two scenarios, the data were sent in the time interval of 1 s in the nonbeacon ZigBee mode. This interval is the same as the high-priority time interval for the EEMIP mode. We have scaled the time interval for low-priority data and we have obtained results for 2 s, 3 s, and 5 s. For all the reported results, we have conducted ten measurements (each taking one hour) and used the average values in the tables. The size of the Acknowledgement frame is constant; 10B according to the specification [25].

### 4.1. Evaluation of Scenario A

The results of the simulation for the nonbeacon ZigBee mode in communication without need for retransmissions are provided in Table 1. The first column represents a device identification number (four devices were simulated). The second column represents the average number of bytes processed by the end device during a simulation time of one hour. The last column represents the average number of packets processed by the end device during the simulation time. One can notice that each device processes the same amount of packets, but different amount of bytes. The reason is that each device produces different amounts of data (device with a higher ID sends more data), which are sent in a single packet.

The results of the simulation for the EEMIP mode in communication without need for retransmissions are provided in Table 2. The table contains analogous values as Table 1; however, it contains results for various low-priority time intervals.

Table 3 contains comparison of results obtained for the two simulation modes. It refers a difference in a number of processed bytes when using EEMIP method in comparison to the standard nonbeacon ZigBee communication.

Based on the reported results for nonproblematic conditions, we can confirm that the EEMIP method sends less data than the standard nonbeacon ZigBee communication in most of the cases (by 8% in average). It means that it is more energy efficient in most of the cases. By lowering the time interval for low-priority data, the data are sent more frequently and the EEMIP method overhead (additional packet headers) is increasing. After some threshold, we can notice that the end devices in the EEMIP mode process more bytes than in the nonbeacon ZigBee mode (i.e., it is less power efficient). The EEMIP method is, thus, beneficial for end devices with a higher amount of data (amount of data is increased with device ID; the end device with the ID of 4 saves the most energy) and with a greater difference between time intervals for low and high priority (when low-priority time interval of 5 s was used, the energy savings were the highest).

### 4.2. Evaluation of Scenario B

In this scenario, there are two kinds of congestions simulated regarding intensity of congestion. It is simulated by a frequency of dropped packets, i.e., how often some data are lost. We simulate a congestion situation, where each third packet is dropped, and the second congestion situation, where each fifth packet is dropped. A dropped packet is simulated by the coordinator not sending the Acknowledgment message. In such a case, retransmission as explained above occurs. This experiment is intended to point-out benefits of the EEMIP method using prioritization.

Table 4 contains the average number of bytes and packets processed by the four end devices during a simulation time of one hour. The results are provided for both congestion situations, where each third or each fifth packet must be retransmitted.

Analogous to Table 4, the results for the EEMIP mode are provided in Table 5 (each 3rd packet must be retransmitted) and Table 6 (each 5th packet must be retransmitted). These tables provide results for three different low-priority time intervals (similar to the previous experiment).

Table 7 contains comparison of results obtained for the two simulation modes for these two congestion situations. It provides a difference in a number of processed bytes and packets when using the EEMIP method in comparison to the standard nonbeacon ZigBee communication.

Based on the reported results for congestion simulation, we can see that the EEMIP method sends more data than the standard nonbeacon ZigBee communication in most of the cases. It might evoke an impression that it is less energy efficient than the standard mode. During a congestion, the coordinator processes the high-priority data first and sends the Acknowledgment message to the end device (which also increases the number of processed bytes). In EEMIP mode, the end devices can transmit data more often without data loss. The data transmission frequency is also increased as a result of no need for delayed waiting for the Acknowledgment message (the mentioned 1.6 s). It means that in the standard ZigBee mode, the end devices more often wait for the Acknowledgment message (upon simulation of data loss) and, thus, effectively transmit less amount of data. To clarify this reasoning, we provide another comparison for the two simulation modes. Table 8 contains a difference in time required for the nonbeacon ZigBee mode to successfully transfer the same amount of data as the EEMIP mode for congestion conditions.

Based on these results, we can confirm that the devices in nonbeacon ZigBee mode have to operate longer than in EEMIP mode to successfully transfer the same amount of data in congested conditions. Since they must operate and communicate longer, they consume more energy. Therefore, this experiment has also shown that the EEMIP mode is more energy efficient than the nonbeacon ZigBee mode.

### 4.3. Evaluation of Scenario C

In this scenario, the data for each packet transmission were generated pseudorandomly, with a size of 1–10 bytes (i.e., a common sensor-data size). The end devices were started one at a time with a delay of 50 ms (i.e., the device 1 was started at a time T, the device 2 was started at a time T + 50 ms, the device 3 was started at a time T + 100 ms, etc.). The high-priority time interval was set to 2 s and the low-priority time interval was set to 10 s. Each end device had both, the low priority data and high priority data. To simulate occasional retransmissions (e.g., interference/collision), each 50th packet was lost. We have conducted three measurements in this experiment (each taking one hour) and used the average values in the reported tables.

Table 9 report the results of such an experiment. The first column represents a device identifier. The second and third columns represent the number of processed bytes and packets, respectively, for the nonbeacon ZigBee simulation. Similarly, the fourth and fifth columns refer the same for EEMIP simulation mode. The last two columns represent the comparison between the two simulation modes.

From the results, we can notice a decrease in the number of processed bytes by 40% on average, when using EEMIP. On the other hand, we can see that the number of transmitted packets is increased by 22%. The number of transmitted bytes is lower in the case of EEMIP because it sends low priority data less frequently. The nonbeacon ZigBee does not deal with priorities, thus, it must send both high and low priority data with the higher frequency. The number of packets is higher in case of EEMIP due to control overhead and the fact that the low priority data are sent in dedicated messages (i.e., separated from the high priority data). Although the number of packets is increased, the reduction in the number of processed bytes is the key indication that the EEMIP method increases energy efficiency in a realistic scenario even more than than previous boundary-cases experiments.

Using the same setup, we have executed another experiment, in which we have scaled the amount of congestion (from occasional to more frequent). This experiment is used to illustrate how the data transmission efficiency is affected by the congestion, comparing nonbeacon ZigBee and EEMIP methods. The data were measured at the coordinator devices (i.e., successfully received messages and lost messages). The measured data are provided in Table 10 and the comparison itself is given in Table 11. The *S_all_* row refers to the processed and sent bytes in the simulated communications. The *R_all_* row represents the amount of bytes successfully received and processed by the receiving device (i.e., without lost messages). The last row (*R_data_*) represents the amount of successfully received bytes of data, i.e., without control overhead.

The result from this experiment is that the EEMIP method successfully transmits a higher amount of useful data by 20% on average during the same simulation time. The result is achieved by eliminating retransmissions of high-priority data, which are more frequent. This increases the communication efficiency, since the parameter of energy per successfully delivered data byte is increased.

Another experiment using the same setup was targeted towards corner cases, in which either all the devices selected the low priority or all the devices selected the high priority for their data. Two congestion situations were simulated. The results of comparisons of EEMIP and nonbeacon ZigBee simulation results are provided in Table 12. The Low priority columns represent the cases in which only the low priority was used, and the High priority columns represent the cases in which only the high priority was used. Other data are represented analogously to Table 11.

From the results, we can see that, although the higher priority data messages could not be preferred over the lower priority messages, the EEMIP method transmitted a higher amount of useful data by 12% in average. In both modes, approximately the same amount of messages were lost. However, in ZigBee, the lost data had to be retransmitted in a separated message, while in EEMIP, the next data message was used (reducing overhead). In most cases, the overhead of nonbeacon ZigBee retransmissions was higher than control overhead of EEMIP. In most cases, the EEMIP mode sends less bytes and also less bytes are successfully received; however, the amount of successfully transmitted useful data were always higher in case of EEMIP (even though insignificantly in case of low priority and 2% packet loss).

### 4.4. Discussion

Based on the experimental results, we can summarize that the proposed method can indeed increase energy efficiency of the ZigBee communication. However, it has also its disadvantages and is limited to specific kinds of communications. The advantages and disadvantages of the proposed method are summarized in Table 13.

The proposed EEMIP method increases energy efficiency (by reducing collisions and retransmissions) for most of the star-based sensor networks using ZigBee, since these are usually periodic, collecting measured data in regular cycles. However, it is not suitable for mostly non-periodic communication (i.e., event based), since it can slightly delay important messages (due to waiting for the time slot). In periodic communication, the waiting time is eliminated using EEMIP. The method uses a control protocol, which increases the overhead. That is why it is unsuitable for occasional communication (e.g., once a day). However, such communication is a domain of other low-power IoT technologies, such as LoRa or Sigfox. The centralized architecture also represents a big downside, since the coordinator represents a single point of failure. However, most of the Internet-connected (via gateway) ZigBee networks have this kind of problem. It can be alleviated in further work by introducing a redundant (grid-powered) coordinator, which would take the role of coordinator in case of failure. The efficiency of the proposed method also depends on the accuracy of the crystal oscillator used in ZigBee devices. Cheap oscillators often suffer from a high inaccuracy, which can result in desynchronization of the end device and the coordinator, and thus disrupt the transmission schedule. In such a case, the coordinator is forced to resend the Offer message to the desynchronized end device, which decreases the energy efficiency if it happens too often. For example, if the end device would send data once a day and due to an inaccurate oscillator it would be received outside the scheduled time slot, the ineffective resynchronization would even increase the energy requirements of such communication. However, the precise boundary point when the proposed method becomes energy efficient depends on the oscillator. It is clear that the resynchronization energy overhead must be lower than the energy benefit gained by the EEMIP method. A rough recommendation is to use the method for communications in which at least 90% of messages are transmitted during the scheduled time slots (i.e., synchronized).

Compared to the existing related works (analyzed in Section 1), the proposed method uses a unique combination of QoS-based prioritization and congestion control and timing channels (duty cycling) to increase communication efficiency. It can be seen as a combination of ideas from different works. Duty cycling [14,15] is a common approach to enable power-saving state of sensor devices; however, we use a unique synchronization mechanism. The medium access incorporating duty cycling to improve energy efficiency can be achieved by modification of link-layer protocol, such as in [13], or it can be influenced by the application protocol as in our case, which eliminates the need for modification of the existing protocol. Proactive planning was used in [16]; however, it was used for Internet connections and we use it to eliminate collisions. A number of works incorporated prioritization into the IoT sphere [18,19,20] to increase reliable delivery of critical communication. However, we have used the priorities in a unique way that also enables us to use them for determination of communication frequency (i.e., periodicity, time intervals). Thus, data with higher priorities are sent more frequently than data with lower priorities.

## 5. Conclusions

In this article, we have presented a new method (called EEMIP) of energy-efficient communication using time intervals and data priority in ZigBee communication for a star network topology. It is most useful for sensor devices, which send collected data periodically. The main contribution is making the communication more energy-efficient while still being effective. The central device in the topology (i.e., coordinator) is aware of all end sensory devices in the network, to which it assigns a time slot for communication and a priority-based time interval. It enables the end devices to communicate in various intervals according to their requirements and eliminates the need to wait for synchronization frames, which wastes the energy. It also handles the congestion, in which it prioritizes the critical data and, thus, ensures reliable delivery. For evaluation of the proposed method, we have implemented a simulator, which was used to compare the standard nonbeacon ZigBee mode and the EEMIP mode. The experimental results confirmed that the EEMIP mode is more energy efficient for most of the cases, in which collisions occur and sensor devices collect various amounts of data with different priorities (often the case of ZigBee networks). For networks without data loss (i.e., less frequent, small amount of devices, environment without interferences, etc.), the proposed EEMIP method is unsuitable due to the increased overhead. Further work can be targeted to the implementation of the EEMIP method in hardware, and evaluating the method using real data.

## Figures and Tables

**Table 1:** Number of processed bytes in the nonbeacon ZigBee mode without retransmissions.

End Device ID	Bytes	Packets
1	55,680	3480
2	76,560	3480
3	97,440	3480
4	118,320	3480

**Table 2:** Number of processed bytes in the EEMIP mode without retransmissions.

End Device	2 s Low-Priority Time Interval		3 s Low-Priority Time Interval		5 s Low-Priority Time Interval	
ID	Bytes	Packets	Bytes	Packets	Bytes	Packets
1	64,140	5040	57,900	4560	52,440	4140
2	78,900	5040	71,220	4560	64,500	4140
3	93,660	5040	84,540	4560	76,560	4140
4	108,420	5040	97,860	4560	88,620	4140

**Table 3:** Comparison of the two simulation modes without retransmissions.

End Device ID	2 s Low-Priority Time Interval	3 s Low-Priority Time Interval	5 s Low-Priority Time Interval
1	+15.19%	+3.99%	−5.82%
2	+3.06%	−6.97%	−15.75%
3	−3.88%	−13.24%	−21.43%
4	−8.37%	−17.29%	−25.1%

**Table 4:** Number of processed bytes in the nonbeacon ZigBee mode during a congestion.

	Each 3rd Packet Lost		Each 5th Packet Lost	
End Device ID	Bytes	Packets	Bytes	Packets
1	29,400	2340	37,440	2700
2	46,920	2460	53,880	2700
3	56,040	2280	69,060	2700
4	69,720	2280	87,300	2760

**Table 5:** Number of processed bytes in the EEMIP mode during a congestion with each 3rd packet lost.

End Device	2 s Low-Priority Time Interval		3 s Low-Priority Time Interval		5 s Low-Priority Time Interval	
ID	Bytes	Packets	Bytes	Packets	Bytes	Packets
1	44,580	3900	51,060	4200	47,940	3960
2	56,220	3840	63,840	4200	58,440	3840
3	68,520	3840	74,100	4080	71,160	3900
4	83,940	3840	85,680	4020	80,340	3780

**Table 6:** Number of processed bytes in the EEMIP mode during a congestion with each 5th packet lost.

End Device	2 s Low-Priority Time Interval		3 s Low-Priority Time Interval		5 s Low-Priority Time Interval	
ID	Bytes	Packets	Bytes	Packets	Bytes	Packets
1	56,160	4560	48,180	4020	48,540	3960
2	66,420	4380	62,100	4080	59,220	3900
3	79,560	4380	72,960	4020	71,580	3900
4	92,880	4380	85,320	4020	84,600	3960

**Table 7:** Comparison of the two simulation modes for congestion conditions.

	Each 3rd Packet Lost			Each 5th Packet Lost		
End Device ID	2 s Low-Priority Time Interval	3 s Low-Priority Time Interval	5 s Low-Priority Time Interval	2 s Low-Priority Time Interval	3 s Low-Priority Time Interval	5 s Low-Priority Time Interval
1	+51.63%	+73.67%	+63.06%	+50.00%	+28.69%	+29.65%
2	+19.82%	+36.06%	+24.55%	+23.27%	+15.26%	+9.91%
3	+22.27%	+32.23%	+26.98%	+15.20%	+5.65%	+3.65%
4	+20.40%	+22.89%	+15.23%	+6.39%	−2.27%	−3.09%

**Table 8:** Time comparison (in minutes) of the two simulation modes for congestion conditions.

	Each 3rd Packet Lost			Each 5th Packet Lost		
End Device ID	2 s Low-Priority Time Interval	3 s Low-Priority Time Interval	5 s Low-Priority Time Interval	2 s Low-Priority Time Interval	3 s Low-Priority Time interval	5 s Low-Priority Time Interval
1	+31	+45	+38	+30	+18	+18
2	+12	+22	+15	+14	+10	+6
3	+14	+20	+17	+10	+4	+3
4	+13	+14	+10	+4	−2	−2

**Table 9:** Comparison of the two simulation modes for a more realistic scenario.

End Device	Nonbeacon ZigBee		EEMIP		EEMIP vs. Nonbeacon ZigBee	
ID	Bytes	Packets	Bytes	Packets	Bytes	Packets
1	20,710	1770	13,020	2150	−37.13%	+21.47%
2	22,480	1760	13,560	2160	−39.68%	+22.73%
3	21,520	1720	12,530	2130	−41.78%	+23.84%
4	20,790	1760	12,620	2130	−39.30%	+21.02%
5	21,560	1740	12,770	2130	−40.77%	+22.41%
6	22,480	1720	13,500	2120	−39.95%	+23.26%
7	21,850	1700	12,260	2100	−43.89%	+23.53%
8	19,990	1730	11,970	2090	−40.12%	+20.81%
9	21,290	1700	12,670	2090	−40.49%	+22.94%
10	20,020	1680	12,310	2080	−38.51%	+23.81%
11	19,970	1670	11,860	2060	−40.61%	+23.35%
12	19,940	1670	12,300	2050	−38.31%	+22.75%
13	19,540	1660	11,500	2010	−41.15%	+21.08%
14	19,170	1650	11,820	1990	−38.34%	+20.61%
15	19,330	1620	12,080	1980	−37.51%	+22.22%
16	19,840	1630	11,730	1960	−40.88%	+20.25%
17	18,550	1610	11,670	1940	−37.09%	+20.50%
18	20,590	1600	11,390	1930	−44.68%	+20.63%
19	20,010	1590	11,390	1920	−43.08%	+20.75%
20	18,900	1560	11,190	1900	−40.79%	+21.79%
**Average**					−40.20%	+21.99%

**Table 10:** Simulation data for a more realistic scenario with various amount of congestion.

	2% Packet Loss		3% Packet Loss		10% Packet Loss		20% Packet Loss	
Bytes	Nonbeacon ZigBee	EEMIP	Nonbeacon ZigBee	EEMIP	Nonbeacon ZigBee	EEMIP	Nonbeacon ZigBee	EEMIP
*S_all_*	317,130	330,680	326,610	339,560	334,260	330,110	320,520	331,670
*R_all_*	315,480	330,080	321,060	338,810	320,910	328,910	297,720	328,070
*S_data_*	115,680	135,300	115,560	140,730	116,910	135,930	108,720	137,490

**Table 11:** Comparison of the two simulation modes for a more realistic scenario with various amount of congestion.

Bytes	2% Packet Loss	3% Packet Loss	10% Packet Loss	20% Packet Loss
*S_all_*	+4.27%	+3.96%	−1.24%	+3.48%
*R_all_*	+4.63%	+5.53%	+2.49%	+10.19%
*S_data_*	+16.96%	+21.78%	+16.27%	+26.46%

**Table 12:** Comparison of the two simulation modes for corner cases.

	2% Packet Loss		20% Packet Loss	
Bytes	Low Priority	High Priority	Low Priority	High Priority
*S_all_*	−5.99%	+6.57%	−11.34%	−2.74%
*R_all_*	−6.15%	+6.57%	−11.76%	−2.98%
*S_data_*	+0.67%	+18.08%	+11.96%	+17.39%

**Table 13:** Advantages and disadvantages of the EEMIP method.

Advantages	Disadvantages
increased energy efficiency	increased control overhead
eliminated processing of periodic beacon frames	limited to star topology
reduced end-device waiting time	single point of failure (coordinator)
reduced number of collisions	benefits limited to frequent periodic communications
reduced number of retransmissions	
increased quality of service	

## Abbreviations

The following abbreviations are used in this manuscript:

IoT	Internet of Things
WSN	wireless sensor networks
BLE	Bluetooth Low Energy
QoS	quality of service
NOMA	NonOrthogonal Multiple Access
TDMA	Time Division Multiple Access
EEMIP	Energy-Efficient Method using Intervals and Prioritization
WPAN	wireless personal area network
